# The Transposon Toolkit for Immune Isoform Diversity in Evolution and Disease

**DOI:** 10.3390/v18101129

**Published:** 2026-10-12

**Authors:** Andrea D. Ordoñez, Edward B. Chuong

**Affiliations:** Department of Molecular, Cellular and Developmental Biology, University of Colorado Boulder, Boulder, CO 80309, USA; anor9792@colorado.edu

**Keywords:** transposable elements, alternative transcription, exonization, isoform diversity, decoy receptors, long-read RNA-seq, species-specific gene regulation, onco-exaptation

## Abstract

Transposable elements (TEs) occupy roughly half of the genomic content across mammals and have long been recognized for their contributions to immune gene regulation as co-opted enhancers and promoters. Less appreciated is their role in diversifying the immune transcriptome itself. TE sequences embedded in introns and gene-flanking regions can provide alternative splice sites, transcription start sites, and polyadenylation signals that generate structurally distinct isoforms of host immune genes. Here we review the mechanisms by which TEs contribute to alternative transcription and the cases with experimental evidence that TE-derived isoforms introduce alternative regulation or function to immune signaling pathways. We give particular attention to the species-specific nature of these events, as each vertebrate lineage carries a distinct TE repertoire and elements shared between species are not always used in the same way, making TE exonization a source of immune regulatory variation both across and within species. These events also contribute to cancer, where epigenetic dysregulation can aberrantly activate TE-derived transcripts with potential as biomarkers and therapeutic targets. Finally, we discuss how advances in long-read sequencing, bioinformatic tools, and proteomics are enabling more systematic characterization of TE-derived isoforms, and outline priorities for cataloguing the full scope of TE contributions to immune transcript diversity.

## 1. Introduction

Immune systems comprise the cellular and molecular networks that detect, respond to, and remember pathogen exposure. The continuous evolutionary conflict between hosts and pathogens makes immune genes among the most rapidly evolving loci across species [1,2,3]. This rapid evolution draws on multiple sources of genetic variation, among which transposable elements (TEs) play a notable role. The genomic occupancy of TEs varies drastically across life, from very low fractions in prokaryotic genomes [4] to over 85% in plants like maize and wheat [5,6]. In vertebrates, TE-derived sequences span roughly 4–60% of assembled genomes, with placental mammals averaging about 50% [7,8,9,10]. This occupancy is not without cost, as new insertions may disrupt coding and regulatory sequences and recombination between copies can promote chromosome rearrangements [11,12,13]. Host genomes therefore deploy dedicated silencing machinery, particularly in the germline [14,15], and purifying selection further depletes TEs from constrained exonic regions, confining most surviving copies to introns and gene-flanking regions [16,17]. Despite these constraints, TEs have made fundamental contributions to the evolution of immune systems across vertebrates.

A prime example is the recombination-activating gene (RAG) machinery that derived from an ancient DNA transposon insertion, an innovation conserved across jawed vertebrates that gave rise to V(D)J recombination and the vast antigen receptor diversity of adaptive immunity [18,19,20]. In a separate but recurring theme, retroviral *gag*- and *env*-derived proteins have been co-opted as antiviral restriction factors that block infection through receptor interference and post-entry restriction [21], including Fv1 in mice [22], endogenous Jaagsiekte sheep retrovirus (enJSRV) in sheep [23], and Suppressyn in humans [24]. Nevertheless, such whole-TE domestications are rare; most TE copies are ancient, degraded remnants with little coding or mobilization potential [25,26,27].

The combination of genomic and transcriptomic studies with chromatin profiling revealed how abundant these degraded sequences are, occupying roughly half of all intronic space in the human genome [28], and that subsets of them are accessible to host transcriptional machinery [28,29]. Early locus-specific studies, followed by genome-wide profiling, demonstrated how TEs can rewire host gene expression when co-opted as regulatory elements, including as enhancers, promoters, insulators, and boundary elements [28,30,31,32,33,34,35,36], and TE-derived regulatory elements have since been found to control innate and adaptive immune genes across species [37,38,39,40,41]. Beyond this cis-regulatory role, TEs also diversify host isoform expression. Through a process broadly termed exonization, TE sequences are incorporated into host messenger RNAs (mRNAs) as novel exons, splice sites (SS), transcription start sites (TSS), or polyadenylation signals (PAS), generating alternative isoforms with the potential to encode functionally distinct proteins.

Early genome annotation and expressed sequence tag (EST)/complementary DNA (cDNA)-based studies provided the first evidence of TE exonization [42,43]. Many of these events were lowly included in mature transcripts or fell within untranslated regions (UTRs), reinforcing the early notion that most TE-derived transcripts were unlikely to substantially alter protein function [17,42,43]. Subsequent cap analysis of gene expression (CAGE) and short-read RNA-sequencing (RNA-seq) studies expanded this view, revealing TE-derived TSS and PAS motifs [29,44] and regulated splice events [45,46,47] that can be tissue- or stimulus-regulated [29,48], aberrantly activated in cancer [47,49], or actively suppressed by host splicing machinery [45,46]. More recently, higher-quality reference genomes, improved repeat annotation, and long-read RNA-seq have enabled systematic identification of TE-derived isoforms [50,51,52,53,54,55], and pairing these with mass spectrometry and ribosome profiling can verify protein output [54,56]. Evidence of function, however, requires perturbing the TE-derived event and measuring a biological effect, and such examples remain limited.

In this review, we focus on the mechanisms by which TEs generate alternative isoforms through exonization across species, with an emphasis on immunity. We summarize the TE families that supply the underlying sequence elements, present characterized examples where TE-derived isoforms alter immune signaling and consider how such isoforms may contribute to differences in pathogen susceptibility and disease risk within and between species. We extend these findings to cancer, where TE-derived transcripts have been implicated as oncogenic drivers, biomarkers, and therapeutic targets, and conclude by discussing how the tools for studying TE contributions to transcript diversity have evolved, along with considerations for future work.

## 2. TE Exonization Contributes to Host Isoform Diversity

The functional complexity of eukaryotic genomes far exceeds what can be explained by gene number alone. A key contributor to this complexity is the alternative transcription of distinct mRNA isoforms from a single genomic locus through the use of alternative SS, TSS, and PAS [57,58,59,60]. In humans, approximately 95% of multi-exon genes undergo some level of alternative splicing (AS) [61], while more than 50% of genes have an alternative TSS and at least 70% contain multiple PAS motifs [58]. All three mechanisms show strong tissue-restricted patterns [58,62,63,64], with isoform identity contributing to tissue-specificity and cell differentiation [65,66], while their misregulation is implicated in a broad range of diseases including cancer, neurological disorders, and autoimmunity [60,67].

**Figure 1:**
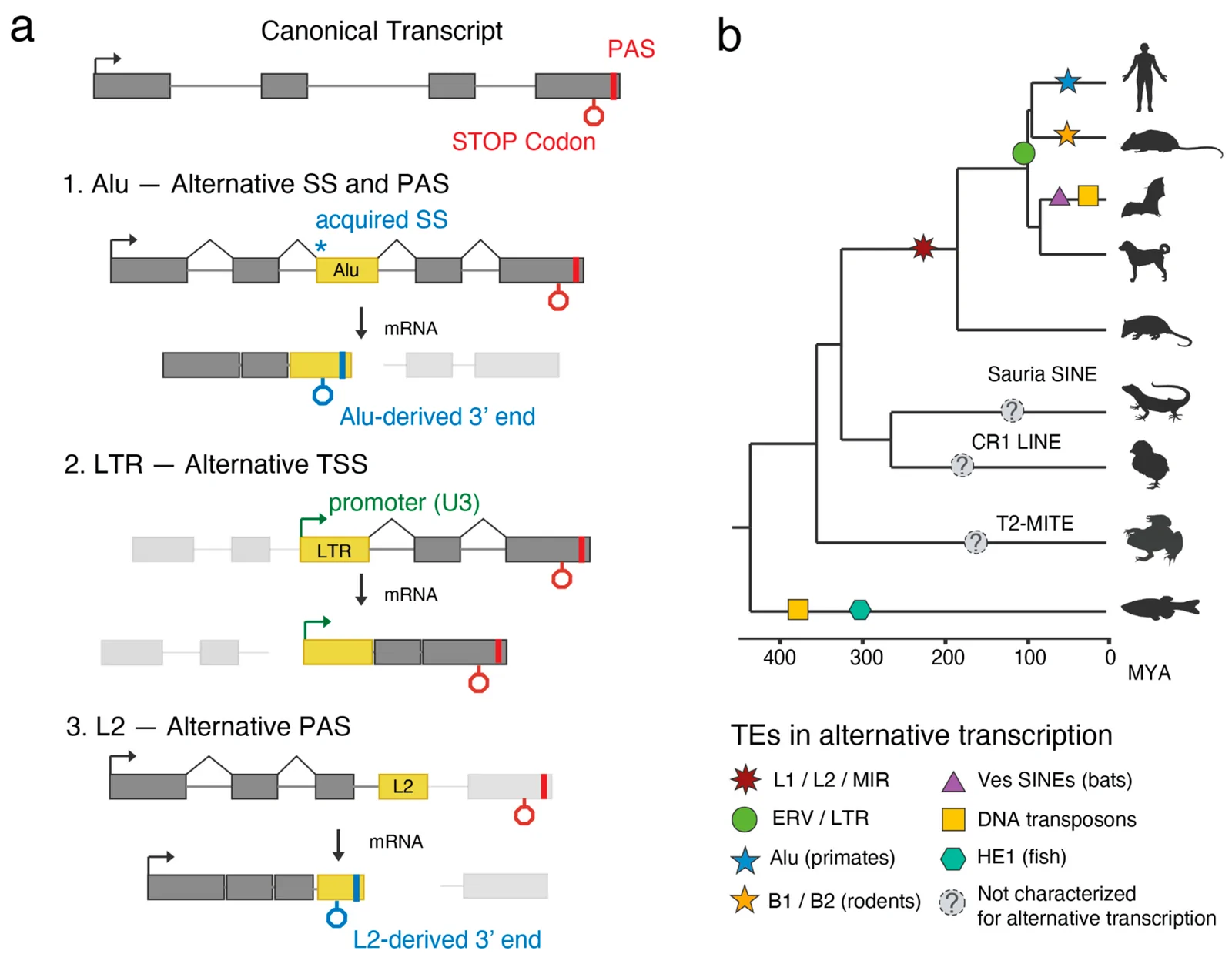
Transposable elements contribute to lineage-specific isoform expression. (**a**) Three mechanisms by which TEs generate alternative isoforms. Top: canonical transcript with host promoter (black arrow), stop codon (open red circle), and polyadenylation signal (PAS, red bar). (1) An Alu element acquires splice sites through mutation (blue asterisk), generating an alternative exon that introduces an Alu-derived 3′ end (open blue circle and blue bar), truncating the protein. Downstream exons are not translated (gray). (2) An LTR element provides a de-repressed promoter (U3 region, green arrow), initiating transcription from an alternative start site. Upstream canonical exons are not transcribed (gray). (3) An L2 element provides an alternative polyadenylation signal within an intron, causing transcriptional read-through and premature termination at an L2-derived 3′ end (open blue circle and blue bar). Downstream exons and the canonical PAS are not included in the mature transcript (gray). Yellow boxes indicate TE-derived sequence incorporated into the mRNA. (**b**) Vertebrate phylogeny highlighting representative TE families implicated in alternative isoform transcription of host genes across major lineages. Colored symbols indicate TE classes or families reported to contribute to alternative transcript architecture. Question-marked symbols indicate lineage-specific TE families for which roles in this mechanism of gene regulation remain uncharacterized. Branch lengths are approximate and shown in millions of years ago (MYA); TE placements are illustrative rather than exhaustive.

TEs near coding regions can introduce novel regulatory sequences for alternative isoform transcription (Table 1). Their genomic distribution shapes these contributions: intronic TEs, which account for approximately 60% of annotated human TE copies, provide abundant substrates for SSs, internal TSSs, and intronic PASs, while insertions in upstream regions, terminal exons, and 3′ UTRs can contribute alternative transcript initiation and termination sites [17,29,61]. Host cells actively suppress TE exonization through various pathways. At the chromatin level, TE silencing represses TE-derived promoters and TSSs, preventing erroneous gene expression [68,69]. At the RNA processing level, RNA-binding proteins including heterogeneous nuclear ribonucleoprotein C (hnRNPC) and scaffold attachment factor B (SAFB) limit TE exonization and prevent premature translational termination from intronic TE sequences [28,45,46]. TE families have entered genomes in successive waves, so any genome contains insertions spanning a wide range of ages. The relative age of an insertion is commonly inferred from its sequence divergence from the consensus sequence of its TE family or subfamily, with more divergent copies generally interpreted as older [9,10]. Surveillance acts unevenly across these elements. Young, relatively intact members of active families, including recently expanded Alu subfamilies, retain sequence features recognized by host machinery and are generally silenced more effectively, although repression is rarely complete. Older copies have accumulated mutations that can erode these features and weaken their recognition [47]. The TE-derived sites that escape suppression can contribute to stable mature transcripts; in this section, we review these mechanisms (Table 1; Figure 1a).

### 2.1. TE-Derived SS

Pre-mRNA splicing depends on conserved motifs at exon-intron boundaries, including 5′ splice donor and 3′ splice acceptor sites, that are recognized by the spliceosome [66,70]. TEs can carry such motifs ancestrally or acquire them after insertion [28,43,45,46]. Endogenous retroviruses (ERVs), for example, derive from retroviruses whose life cycles depend on host splicing [64,71]. However, most ERV copies have been reduced to solo long terminal repeats (LTRs), so their principal contribution comes through alternative TSS and PAS motifs, discussed in the following section.

Other TE families carry cryptic SSs, which become active through several routes. The most direct is mutation within the element, where single nucleotide changes create a functional site and the surrounding sequence determines which of several competing sites is selected for splicing [57,67] (Figure 1a). Recognition also depends on the trans-acting environment, best illustrated by Alu elements. These primate-specific dimeric short interspersed nuclear elements (SINEs) carry abundant cryptic SSs on their antisense strand [43,57,68] and contribute more to exonization than any other TE family in the human genome [17,53]. This burden is met by a dedicated layer of surveillance: hnRNPC binds these sites and competes with U2 small nuclear RNA auxiliary factor 65 (U2AF65) for SS recognition. Depletion of hnRNPC causes massive Alu exonization across cellular transcriptomes [45,46]. Activation of a cryptic SS rarely occurs in a single step. These sites typically begin as weak signals used at low levels, and are progressively strengthened by successive mutations [46,57,67]. Their use is further modulated by chromatin state and transcriptional elongation rates, which can favor weak splice sites [72,73]. Sequence features within the element further tune inclusion. Alu’s dimeric architecture imposes one such layer: the intronic left arm carries weaker signals than the exonized right arm and acts in cis to keep these exons alternatively rather than constitutively spliced. This tempered inclusion allows Alu-derived exons to persist rather than be selected against [46,68]. Mammalian-wide interspersed repeat (MIR) elements act through a different feature, carrying exonic splicing enhancers that strengthen recognition of a newly acquired SS [59,60].

Mutation-driven acquisition of SSs and modulation by trans-acting factors are general mechanisms, but their consequences depend on the sequence of the element involved, as the Alu examples show. Other families carry their own distinctive attributes, and with so few characterized in detail, the mechanisms by which TEs contribute to AS remain largely unknown. Most of this evidence is also transcript-level. Detection of a TE-derived isoform, even one that is tissue-regulated or conserved, does not establish that it yields a functional product. Many spliced TE exons introduce premature stop codons (Figure 1a) that target the transcript for nonsense-mediated decay (NMD) [46,56], modulating protein levels without altering protein structure. Resolving which events produce functionally distinct isoforms requires proteomic or perturbation approaches, for which there are few examples.

### 2.2. TE-Derived TSS and PAS

Alternative transcript initiation and termination sites are the principal drivers of tissue-specific isoform expression in humans, exceeding the contribution of AS [74]; TEs are an important source of both (Figure 1a; Table 1). Compared with TE-derived AS, which often depends on the activation or strengthening of latent splice-like motifs, TE co-option at the level of TSS or PAS commonly draws directly from regulatory features present in the ancestral TE sequence [58,61].

Among TE families contributing to alternative TSS, LTR retrotransposons [64,65] and LINEs [29,63] are best characterized. The long terminal repeats of LTR retrotransposons carry promoter sequences (Figure 1a) that drive transcription during the retroviral life cycle [64,65], while L1 LINEs harbor a bidirectional promoter in their 5′UTR whose antisense orientation can drive transcription of adjacent host genes, generating chimeric transcripts [29,63]. In a recent multi-omic survey across 40 human tissues and embryonic stem cells, Zhang et al. identified 14,164 TE-initiated transcripts arising from 14,305 TE loci; LTRs were the largest contributor (5642 loci; ~39%), followed by LINEs (3855; ~27%) and SINEs (3176; ~22%), and 80% of TE-initiated transcripts were tissue-specific [75].

At the 3′ end of transcripts, diverse TE families can contribute functional PAS motifs that promote premature or alternative transcript termination, including LTRs, LINEs, SINEs, and DNA transposons [55,58,61,64] (Figure 1a; Table 1). Conley and Jordan identified more than 9000 TE-derived termination sites used by 5747 human genes across eight ENCODE cell types and found these sites to be more cell-specific than canonical termination sites [44]. Comparative analysis across human, mouse, rat, chicken, and zebrafish further showed that non-conserved PAS motifs are disproportionately TE-associated [61]. Importantly, only a small fraction of these are likely to be functional transcript termination signals because productive 3′ end formation depends on local sequence context and processing efficiency, not simply the presence of a PAS hexamer motif [55,58,61].

Several considerations specific to TSS and PAS shape how confidently these events can be identified and interpreted from RNA-seq data alone. Unlike SSs, whose recognition is defined by well-established consensus sequences at exon-intron boundaries, assigning promoter activity to TE sequences found at the 5′ ends of transcripts is more ambiguous and often requires additional evidence such as chromatin accessibility, promoter-associated histone marks, or transcription factor binding motifs [29,76,77]. Similarly, designating a TE-derived PAS hexamer as a functional termination signal requires evidence of 3′-end processing or transcript termination [44,55,58,61]. Additionally, several foundational studies characterizing TE-derived TSS or PAS relied on cell line datasets [29,44], where epigenetic derepression may unmask TEs silenced in primary tissues; including such contexts is valuable for discovery but warrants careful interpretation. As with TE-derived AS, whether detected alternative TSS and PAS events generate functional protein products remains largely unresolved and is an important gap to address.

## 3. Species-Specific Immune Adaptations from TE-Derived Isoforms

Vertebrate genomes carry distinct TE repertoires [48,62,78], so TE exonization can generate species-specific isoforms (Figure 1b; Table 1). Examples are well established in mammals, including Alu-derived human isoforms [17,57], B1 SINE-derived mouse isoforms [17], and isoforms arising from lineage-specific LTRs [48,79]; since retroviral infection and germline transmission continue to generate new endogenous retroviral insertions, LTR exonization remains an ongoing source of lineage-specific transcript innovation [64,80]. Immune genes are among the most rapidly evolving loci in vertebrate genomes [1,2,3] and are increasingly recognized as having been shaped by TEs [37,38,39,40,41]. Consistent with this, transcriptomic studies have cumulatively identified thousands of TE-derived transcript events in immune and inflammatory genes across mammals, many of them species-specific [48,53,54,55,79,81].

Most of these events fall in UTRs rather than coding sequence [17,42,43,82], where they can introduce alternative TSSs and promoters at the 5′ end [29,63,75] or alternative termination sites and sequences affecting transcript stability at the 3′ end [44,58,61,81]. Such events alter how much of a protein is made without changing the protein itself. TE exonization that alters the protein-coding sequence has been comparatively underappreciated, reflecting both purifying selection against disruptive insertions in coding regions [16,17,81] and the methodological limits discussed in Section 5. In this section we focus on these events, prioritizing those with proteomic and functional evidence that the isoform yields a protein with altered activity.

One way TEs contribute to immune gene regulation is by providing alternative promoters and TSSs that place host genes under stimulus-responsive control. The consequences range from changes in expression alone to a novel first exon that alters the encoded protein. In primary human CD4 T cells, human immunodeficiency virus type 1 (HIV-1) infection activates LTR12C elements that drive expression of the antiviral genes guanylate-binding protein 2 (GBP2) and guanylate-binding protein 5 (GBP5) [83]. A comparable pattern was predicted from transcriptomic data in interferon (IFN)-stimulated *Myotis lucifugus* cells, including a bat-specific LTR39B2_ML element at NLRC5, though these events have not been experimentally validated [48]. In both cases the coding sequence is untouched. At the Angiotensin-Converting Enzyme 2 (ACE2) locus, by contrast, intronic MIRb and LTR16A1 retroelements function as an IFN-responsive alternative promoter and novel first exon, generating a truncated isoform, dACE2/MIRb-ACE2, that is strongly induced by interferons while canonical ACE2 is largely unresponsive [84,85]. The truncated protein lacks both the severe acute respiratory syndrome coronavirus 2 (SARS-CoV-2)-binding residues and the carboxypeptidase activity of canonical ACE2, rendering it non-functional as a receptor and as an enzyme [84,85] (Table 2). Whether dACE2 has other regulatory roles in ACE2 signaling remains to be studied, but establishing that it is non-functional resolved early concerns that IFN-based coronavirus disease 2019 (COVID-19) therapies might upregulate the viral entry receptor.

**Figure 2:**
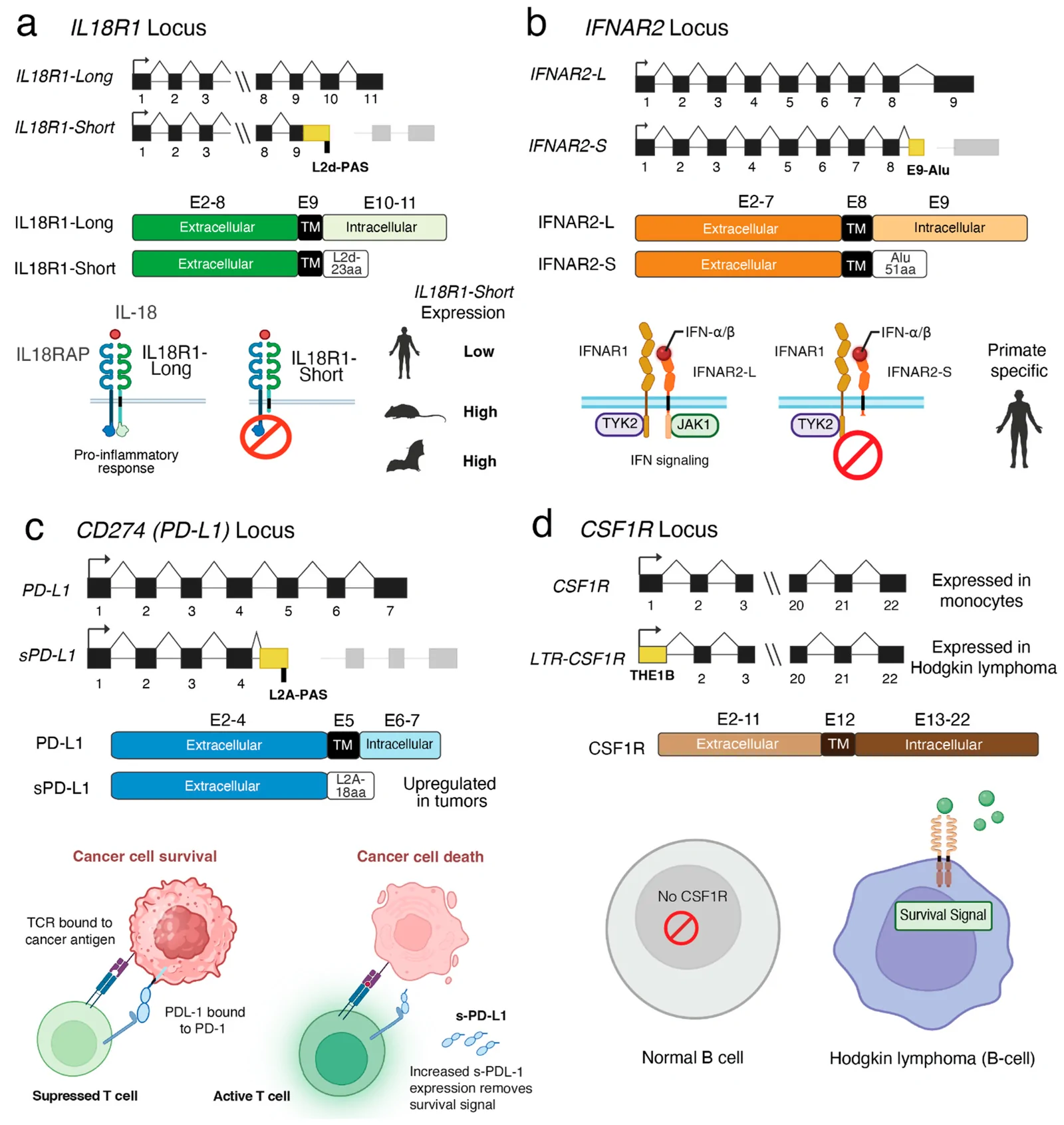
TE-derived alternative isoforms at immune gene loci. Each panel shows transcript structure (**top**), protein domain architecture (**middle**), and functional consequence or expression context (**bottom**). Bent arrows indicate transcription initiation sites; black boxes represent host-derived exonic sequences, yellow boxes indicate TE-derived sequences, and gray boxes show canonical exons excluded from the isoform; double slashes indicate omitted exons, and vertical black bars mark TE-derived polyadenylation signals; red circle-and-slash symbols indicate absent signaling in panels a and b and absent CSF1R expression in panel d. E, exon; TM, transmembrane domain; aa, amino acids. (**a**) An L2-derived polyadenylation signal (L2d-PAS) in intron 9 of IL18R1 generates a truncated isoform (IL18R1-Short) lacking the intracellular TIR signaling domain. IL18R1-Short binds IL-18 but cannot recruit downstream signaling adaptors, functioning as a decoy receptor that dampens pro-inflammatory signaling. IL18R1-Short is the dominant isoform in epithelial barrier tissues of mouse and bat but not human. (**b**) A primate-specific Alu-Jr element provides an alternative terminal exon (E9-Alu) at the IFNAR2 locus, replacing canonical exon 9 and truncating the intracellular domain. IFNAR2-S retains the extracellular FNIII domains and binds IFN-α/β with IFNAR1 but cannot recruit JAK1, functioning as a decoy receptor that inhibits type I IFN signaling. IFNAR2-S is the dominant isoform in nearly all human tissues. (**c**) Exonization of a LINE-2A element in intron 4 of CD274 drives alternative polyadenylation, generating soluble PD-L1 (sPD-L1) that retains the extracellular Ig-like domain but lacks the transmembrane and intracellular domains. Membrane-bound PD-L1 engages PD-1 on T cells to suppress anti-tumor immunity; sPD-L1 acts as a receptor antagonist that blocks this interaction, restoring T cell activation. sPD-L1 is upregulated in tumors. (**d**) Aberrant activation of a THE1B MaLR LTR drives lineage-inappropriate expression of full-length CSF1R in Hodgkin lymphoma. CSF1R is normally expressed in monocytes from its canonical promoter but silenced in B cells. In B cell-derived Hodgkin lymphoma, THE1B de-repression produces a chimeric LTR-CSF1R transcript encoding the same protein, resulting in CSF1-dependent survival signaling.

Loss of protein-coding domains, like in the ACE2 example, is a common theme of TE-exonization. In many cases, these truncated products tend to generate signaling-defective or decoy receptor variants that commonly modulate pathways and introduce immune regulation (Figure 2; Table 2). At the Interleukin 18 Receptor 1 (*IL18R1*) locus, an ancient LINE-2-derived polyadenylation signal generates a truncated decoy (IL18R1-Short) that dampens IL-18/nuclear factor kappa B (NF-κB) pro-inflammatory signaling. *IL18R1-Short* is robustly expressed in bat and mouse tissues, particularly at epithelial-barrier contact sites, but is lowly expressed across human tissues, highlighting species-specific utilization of the same ancestral TE [55] (Figure 2a). At the Interferon Alpha and Beta Receptor Subunit 2 (*IFNAR2*) locus, a primate-specific Alu provides an alternative terminal exon producing a truncated decoy receptor (IFNAR2-S) that inhibits type I IFN signaling and is more expressed than the canonical isoform across the majority of human tissues [53] (Figure 2b). At the Interleukin 13 Receptor Alpha 1 (*IL13RA1*) locus, primate-specific retrotransposable elements provide an alternative terminal exon that removes critical cytoplasmic signaling domains, and the resulting isoform antagonizes canonical IL-4 and IL-13 receptor function [86] (Table 2).

TE exonization can alter protein architecture not only through truncation, but also by introducing internal coding sequence. An Interleukin 15 Receptor Subunit Alpha (IL15RA) isoform contains a TE-derived exon predicted to introduce two α helices between the signal peptide and the IL-15-binding Sushi domain, potentially modifying receptor–ligand interactions while preserving the remainder of the receptor [54]. Although the isoform reaches the plasma membrane when ectopically expressed, its endogenous protein production and the functional consequences of the insertion remain unestablished [54]. Clear examples come from non-immune genes. In thymopoietin (TMPO), a DIRS1-like retrotransposon supplies the unique C-terminal domain of lamina-associated polypeptide 2α (LAP2α), which helps organize the nucleus and regulate cell-cycle progression [93]. In the SET domain and mariner transposase fusion gene (SETMAR), an Hsmar1-derived exon supplies the transposase domain. Although it no longer supports efficient transposition, this domain retains DNA-binding and other ancestral transposase activities [94,95]. Comparable functional domains derived from TE sequence have not yet been established in immune proteins, although the IL15RA isoform represents a plausible candidate.

Across the examples above, demonstrating function has required combining isoform-specific knockdown, knockout, or overexpression in relevant cell types with biochemical assays of receptor binding or downstream signaling, alongside expression profiling to establish physiological context (Table 2). These approaches have shown that TE-derived isoforms can modulate immune signaling, but two gaps remain. Proteomic validation that detected transcripts yield stable protein products is lacking for most examples, and functional characterization has concentrated in primates, rodents, and to a lesser extent bats, leaving the broader vertebrate landscape untested.

## 4. TE-Derived Immune Isoforms in Health and Disease

Species differ in how they respond to the same pathogen, and TE-derived isoforms are one potential source of such variation. Bats tolerate viruses that cause damaging inflammation in humans [96]. As described above, IL18R1-Short dampens signaling by the proinflammatory cytokine IL-18 and is highly expressed in bat epithelial barrier tissues while expressed at low levels in human tissues [55] (Figure 2a). Testing whether this decoy receptor contributes to differences in inflammatory response will require comparing IL-18 responses in bat and human cells under matched conditions, with isoform-specific perturbation.

Within species, natural variation at TE exonization events can modulate individual disease risk. Genome-wide association study (GWAS) variants associated with severe COVID-19 are located in and near the Alu-derived exon E9-Alu of IFNAR2 [53], raising the possibility that polymorphism at this exon modulates decoy receptor expression and therefore individual capacity to regulate type I IFN signaling. Establishing this will require patient-level data connecting specific variants to E9-Alu expression, alongside perturbations testing whether altered decoy levels change IFN signaling output. The primate-specific IL13RA1-LOR1a isoform is estrogen-regulated and sexually dimorphic in expression, antagonizing IL-4 and IL-13 receptor signaling [86]. Its disease relevance remains untested, but it offers a TE-derived mechanism linking sex-biased cytokine receptor regulation to allergic inflammation and IL-4/IL-13-associated tissue remodeling and fibrosis [86,97,98,99].

In cancer, systematic surveys have identified over 100 oncogenes driven by TE cryptic promoter activation across 15 cancer types [47,49,100,101] (Table 2). Unlike the co-option events described above, which were selected for and maintained as functional regulators, cancer-associated TE-derived transcripts typically arise from aberrant activation of normally silenced TE promoters or from tumor-associated disruption of transcript processing [49,100,101]. Among immune genes, the best-characterized case is Colony-Stimulating Factor 1 Receptor (CSF1R), where an aberrantly activated MaLR LTR drives lineage-inappropriate expression in Hodgkin lymphoma that is central to tumor cell survival [88] (Figure 2d). This is one of the few examples with a demonstrated causal role in tumorigenesis; most remain correlative, though their cancer specificity makes them candidate biomarkers or therapeutic targets pending clinical validation. A distinct therapeutic angle involves deliberately un-silencing cryptic TE promoters with epigenetic therapies, using DNA methyltransferase inhibitors (DNMTi) or histone deacetylase inhibitors (HDACi) to generate chimeric transcripts and exon-TE junction antigens that can be immunogenic and elicit anti-tumor T cell responses [102,103,104]. Recurrence of individual TE-derived isoforms across patient populations remains poorly characterized, however, and the lack of selectivity in epigenetic activation risks genomic instability and off-target effects.

## 5. Bioinformatic and Multiomic Approaches for TE-Derived Isoform Discovery

TE-derived isoforms can be expressed at low levels and restricted to particular tissues, cellular states, or species, making their identification challenging [52,53,54,55]. Their discovery therefore depends on broad sampling, high-quality reference genomes and gene annotations, and sequencing data that can resolve transcript structure. Short-read RNA-seq provides substantial depth but reconstructs isoforms from fragmented reads. Long-read sequencing can instead span complete transcripts and anchor alternative exons or transcript ends in unique flanking sequence [50,51]. Long-read workflows have been used to resolve copy-specific TE transcription and splicing [52], identify TE-derived immune isoforms across tissues and species [53,55], and map TE–gene chimeras more broadly [79]. Combining platforms is often most effective, with long reads defining isoform structure and short reads providing depth for quantification [52,55].

Determining whether a reconstructed isoform is TE-derived requires repeat annotations, generated by comparison with curated consensus libraries such as Dfam and often supplemented by de novo repeat discovery [9,10,105], followed by intersection of transcript models with TE coordinates [44,49,53,54]. This remains a bottleneck in comparative work because many species lack high-quality assemblies or well-curated repeat libraries. With short reads, repetitive TE sequences also create multimapping reads, which may be excluded, aggregated by family, or assigned probabilistically [106,107,108]. TEtranscripts and SQuIRE support family- and locus-level TE quantification [52,108], whereas isoform discovery requires evidence connecting the TE to a host transcript. TEProF2 identifies TE–gene fusion transcripts and predicts their coding potential [100]. For TE-derived splice sites, junction-spanning reads directly demonstrate inclusion in a mature transcript, as implemented in the JET workflow [54,103] and related tumor catalogues [104]. For TE-derived TSSs and PASs, assignment depends on transcript boundaries, read coverage, and element position. LIONS detects TE-initiated transcripts from short-read data [90], while long-read isoforms can be intersected directly with Dfam or RepeatMasker annotations, as in Pasquesi et al. and Ordonez et al. [53,55]. Dedicated 5′- and 3′-end sequencing provides additional support for capped start sites and cleavage and polyadenylation sites [44,49,61,102].

Transcript detection does not establish translation, and TE-derived transcripts are extensively regulated post-transcriptionally: premature stop codons target many for NMD [79], A-to-I editing by adenosine deaminase acting on RNA (ADAR) enzymes can create or disrupt splice signals in Alu-containing transcripts [109,110], and N6-methyladenosine (m6A) modification contributes to silencing of retrotransposon-derived transcripts [111,112,113]. Ribosome profiling can identify translated TE-derived regions and mass spectrometry can detect peptides unique to the TE-derived isoform [54,56]. Both provide strong evidence of protein production, although isoform-specific detection remains difficult for low-abundance transcripts. However, protein-level evidence still does not establish what an isoform does. A truncated product may act as a decoy, be secreted, be degraded, or have no consequence, while a TE-derived coding insertion may alter localization, stability, or ligand interaction. Sequence and abundance cannot distinguish these outcomes, so isoform-specific perturbation followed by signaling or reporter assays remains the standard for functional characterization, as illustrated in Section 3. Such evidence exists for a few TE-derived immune isoforms [53,54,55], but expanding discovery across samples, tissues, and species will supply candidates for testing.

## 6. Outlook and Perspectives

Immune systems are a context in which TE co-option has repeatedly given rise to innovation. As our ability to interrogate genomic, transcriptomic, and proteomic content has advanced, we have come to appreciate how extensive TE insertions are near coding regions and how frequently they are used to regulate host gene expression. This review focused on one mechanism, TE exonization into mature mRNA transcripts, which is conserved across species and can generate protein variants with consequences for immune function and regulation. That such events persist at all is notable, given that TE activity is continuously surveilled and restricted, and we are only beginning to understand the range of ways TEs facilitate evolution under those constraints.

Multiple pipelines now exist for genome-wide discovery of TE-derived isoforms, yet much remains to be catalogued. These events can be rare, so their detection requires repeated sampling across diverse contexts. Cross-species atlases built from long-read RNA-seq have improved our ability to capture lineage-specific immune transcript diversity, but have been applied to only a handful of species, nearly all of them mammals. Extending this work to other vertebrates could uncover TE families with distinct mechanisms of exonization. Cross-tissue atlases are equally important given the strong tissue-specificity of TE-derived isoform expression, and cross-disease atlases spanning autoimmunity, chronic inflammation, infection, and cancer would help clarify which isoforms are clinically actionable.

Few studies have moved past discovery into proteomic evidence of translation and its functional consequences, which is what distinguishes TE-derived isoforms that contribute to adaptation from those that are tolerated genomic noise. The examples in Section 3 and Section 4 are convincing on this point, but they are few against the plethora of TE-derived isoforms now identified. Progress will require systematic prioritization of the isoforms most likely to matter, including those that are highly expressed, supported by protein-level evidence, and situated in key immune pathways.

These efforts also carry translational potential. Each species harbors TE-derived immune adaptations shaped by millions of years of host–pathogen coevolution, and characterizing them across vertebrates could reveal regulatory strategies for tuning immune responses rather than simply activating or silencing them. In cancer, the epigenetic dysregulation of tumor cells can aberrantly activate TE-derived transcripts that are absent or far less abundant in matched normal tissue, making them tumor-enriched features that may serve as biomarkers, therapeutic targets, or predictors of treatment response. Realizing either possibility will require connecting what has so far been studied separately, namely the evolutionary origins of TE-derived isoforms, their molecular mechanisms, and their roles in disease, so that the field can move from individual case studies toward a systematic account of how TEs shape immune transcript diversity.

## Figures and Tables

**Table 1:** TEs involved in alternative transcription.

TE Family/Class	Evolutionary Context	Observed Sequence Contribution
Alu/SINE	Young primate SINE derived from 7SL RNA; currently active via L1-dependent mobilization.	SS, PAS, TSS [29,43,57,58]
MIR/SINE	Ancient mammalian tRNA-derived SINE; inactive.	SS, PAS [17,59,60,61]
B1 and B2/SINE	Young rodent SINEs (B1 from 7SL RNA, B2 from tRNA); currently active via L1-dependent mobilization.	SS, PAS [17,61]
HE1/SINE	Ancient tRNA-derived SINE found in fish and sharks; inactive.	SS [62]
SVA/composite retrotransposon	Young hominoid composite element (SINE-VNTR-Alu); currently active via L1-dependent mobilization.	SS, PAS [11]
L1/LINE	Young-to-ancient mammalian LINE; <0.05% of copies remain retrotransposition-competent in humans.	TSS, PAS, SS [17,29,58,63]
L2/LINE	Ancient mammalian LINE; inactive.	PAS, SS, TSS [17,29,55,61]
ERVs/LTR retrotransposon	Ancient-to-young retroviral-derived elements; inactive in most species, with most genomic copies degraded to solo LTRs.	TSS, PAS [58,61,64,65]
DNA transposons	Ancient in most mammals; active in vespertilionid bats and teleost fish.	SS, PAS [61,62]

SS = splice site; TSS = transcription start site/promoter; PAS = polyadenylation signal. Listed contributions reflect reported mechanisms and do not exclude additional functions. “Active” denotes families with copies that remain transposition-competent in the indicated species.

**Table 2:** TE-derived isoforms in immunity and disease.

Gene/Isoform	Species	TE Element	Sequence Contribution	Protein Consequence	Functional Evidence
Evolutionary Co-option					
IFNAR2/ IFNAR2-S [53]	Human	Alu	SS + PTC	C-term: signaling domain lost	IFNAR2-S predominant in most tissues; decoy, KO ↑IFN signaling; CRISPR deletion of TE ↓IFNAR2-S.
IL18R1/ IL18R1-Short [55]	Eutherian and marsupial mammals	L2d	PAS	C-term: signaling domain lost	IL18R1-Short high in bat/mouse barrier tissues, low in human tissues; decoy, KD ↑IL-18 signaling; TE PAS motif analysis and conservation across species.
ACE2/ dACE2 [84,85]	Human	MIRb/ LTR16A1	TSS/ promoter	N-term: catalytic + viral binding residues lost	dACE2 low basal, IFN-inducible in airway/GI; non-functional, no spike binding or enzyme activity; 5′-end mapping to TE promoter.
IL13RA1/ IL13RA1-LOR1a [86]	Human	LOR1a/ L1MD1	SS + PTC	C-term: cytoplasmic signaling domains lost	IL13RA1-LOR1a low-moderate; partial signaling defect, antagonizes IL-4/IL-13; CRISPR deletion of TE ↓IL13RA1-LOR1a.
CD274/ sPD-L1 [87]	Human	L2a	PAS	TM domain lost; novel 18-aa C-term	sPD-L1 low in normal tissues, upregulated in tumors; soluble PD-1 antagonist, no T cell inhibition; TE deletion in minigene ↓sPD-L1.
Cancer-Associated					
CSF1R/ THE1B-CSF1R [88]	Human (HL, ALCL)	THE1B LTR	TSS/ promoter	Full-length; TE-derived 5′ UTR	THE1B-CSF1R in HL, absent from normal B cells; CSF1R blockade ↓HL proliferation; bisulfite pyrosequencing shows THE1B hypomethylation/derepression in HL.
SP140/ LTR8B-SP140 [89]	Human (multiple cancers)	LTR8B	TSS/ promoter	Full-length; TE-derived 5′ UTR	LTR8B-SP140 recurrent in non-immune cancers; CRISPRi of LTR8B ↑IFNβ sensitivity; CRISPRi of LTR8B ↓LTR8B-SP140 (−88%).
IL33/ LTR-IL33 [90]	Human (CRC)	MSTD LTR	TSS/ promoter	N-term: regulatory domain lost	LTR-IL33 in 21/66 CRCs, absent from normal colon; KD ↓colonosphere growth; 5′RACE maps to MSTD LTR.
ERBB4/ LTR-ERBB4 [91]	Human (ALK− ALCL)	Intronic LTRs	TSS/ promoter	N-term: ectodomain lost (I20Δ, I12Δ)	LTR-ERBB4 in ~24% ALK− ALCL; ERBB4 inhibition ↓ALCL growth; 5′RACE maps to intronic LTR.
FABP7/ LTR2-FABP7 [92]	Human (DLBCL)	LTR2	TSS/ promoter	Altered N-term sequence	LTR2-FABP7 in a subset of DLBCL, absent from normal B cells; KD ↓proliferation; bisulfite sequencing shows LTR2 hypomethylation/derepression in DLBCL.
LIN28B/ LIN28B-TST [49]	Human (HCC, other cancers)	AluJb	TSS/ promoter	Full-length; TE-derived 5′ UTR	AluJb-LIN28B tumor-associated and placenta-expressed, poor prognosis in HCC; AluJb-LIN28B ↑proliferation and tumor growth; CRISPR deletion of AluJb ↓AluJb-LIN28B.

Gene names not defined in the main text: SP140, Speckled Protein 140; IL33, Interleukin 33; ERBB4, Erb-B2 Receptor Tyrosine Kinase 4; FABP7, Fatty Acid Binding Protein 7; LIN28B, Lin-28 Homolog B; CD274, Programmed Death-Ligand 1 (PD-L1). Abbreviations: SS, splice site; PAS, polyadenylation signal; TSS, transcription start site; PTC, premature termination codon; TM, transmembrane; C-term, C-terminal; N-term, N-terminal; KO, knockout; KD, knockdown; CRISPRi, CRISPR interference; 5′RACE, rapid amplification of cDNA ends; HL, Hodgkin lymphoma; ALCL, anaplastic large-cell lymphoma; DLBCL, diffuse large B-cell lymphoma; HCC, hepatocellular carcinoma; CRC, colorectal cancer; GI, gastrointestinal. Symbols: ↑ and ↓ indicate increases and decreases, respectively, in the indicated expression level, signaling response, sensitivity, proliferation, or growth.

## Data Availability

No new data were created or analyzed in this study. Data sharing is not applicable to this article.
